# Seroprevalence of Rodent Pathogens in Wild Rats from the Island of St. Kitts, West Indies

**DOI:** 10.3390/ani9050228

**Published:** 2019-05-10

**Authors:** Kenneth Boey, Kanae Shiokawa, Harutyun Avsaroglu, Sreekumari Rajeev

**Affiliations:** Ross University School of Veterinary Medicine, P.O. Box 334, Basseterre, St. Kitts, West Indies; boey_kenneth@hotmail.com (K.B.); kshiokawa@yahoo.com (K.S.); havsaroglu@rossvet.edu.kn (H.A.)

**Keywords:** seroprevalence, rodents, rats, pathogens, laboratory, biosecurity, St. Kitts, Caribbean

## Abstract

**Simple Summary:**

The role of rodents in the transmission of many diseases is widely known. Wild rats abundant in urban environments may transmit diseases to humans and other animals, including laboratory rodents used for biomedical research in research facilities, possibly compromising research data. In order to gather information about the various diseases present around such facilities, it is important to conduct routine surveillance of wild rodents in the area. In this pilot study, we surveyed 22 captured wild rats (*Rattus norvegicus* and *Rattus rattus*) from the Caribbean island of St. Kitts for 19 microorganisms. Information gained from such surveillance data would be beneficial in assessing regional public health risks and when implementing routine laboratory rodent health monitoring protocols.

**Abstract:**

A pilot seroprevalence study was conducted to document exposure to selected pathogens in wild rats inhabiting the Caribbean island of St. Kitts. Serum samples collected from 22 captured wild rats (*Rattus norvegicus* and *Rattus rattus*) were tested for the presence of antibodies to various rodent pathogens using a rat MFI2 serology panel. The samples were positive for cilia-associated respiratory bacillus (13/22; 59.1%), *Clostridium piliforme* (4/22; 18.2%), *Mycoplasma pulmonis* (4/22; 18.2%), *Pneumocystis carinii* (1/22; 4.5%), mouse adenovirus type 2 (16/22; 72.7%), Kilham rat virus (15/22; 68.2%), reovirus type 3 (9/22; 40.9%), rat parvovirus (4/22; 18.2%), rat minute virus (4/22; 18.2%), rat theilovirus (2/22; 9.1%), and infectious diarrhea of infant rats strain of group B rotavirus (rat rotavirus) (1/22; 4.5%). This study provides the first evidence of exposure to various rodent pathogens in wild rats on the island of St. Kitts. Periodic pathogen surveillance in the wild rat population would be beneficial in assessing potential regional zoonotic risks as well as in enhancing the current knowledge when implementing routine animal health monitoring protocols in facilities with laboratory rodent colonies.

## 1. Introduction

Wild and peridomestic rats, especially the Norway rat (*Rattus norvegicus*) and black rat (*Rattus rattus*), are known reservoirs of a number of rodent and zoonotic pathogens [1]. They are ubiquitous in urban and rural environments and are major pests of public health significance, as they carry and transmit pathogens that can cause significant mortality in humans and animals [2]. Wild rats may pose an animal biosecurity risk to laboratory rodent colonies due to inadvertent transmission, possibly causing significant complications in biomedical research [3], in addition to zoonotic risks to laboratory animal caretakers and other personnel.

To gather pertinent information and document evidence of the exposure to common rodent pathogens in wild rats inhabiting the island of St. Kitts, West Indies, we conducted a pilot seroprevalence study and screened wild rats from the island for selected rodent pathogens.

## 2. Materials and Methods

This study was conducted at Ross University School of Veterinary Medicine (RUSVM), Basseterre, St. Kitts, West Indies, adhering to a protocol approved by the Institutional Animal Care and Use Committee (protocol no. 17-01-04).

### 2.1. Wild Rat Trapping

**Figure 1:**
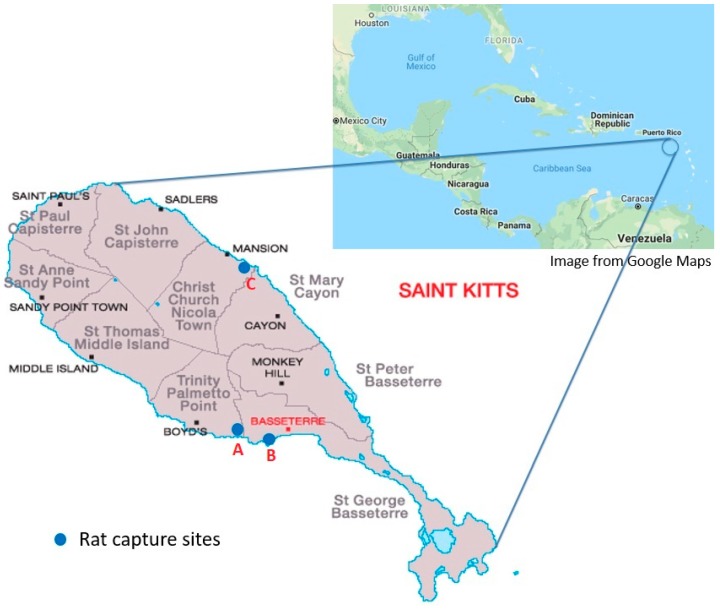
Map of St. Kitts, West Indies [4], showing the parishes and areas of rat capture sites (blue circles). A, around the campus of Ross University School of Veterinary Medicine in West Farm, and residential dwellings in Mattingley; B, near commercial establishments in downtown Basseterre and Port Zante; C, around the campus of the St. Kitts Biomedical Research Foundation (nonhuman primate facility) in Lower Bourryeau Estate.

Wild rats were captured with live traps (Tomahawk Trap Co, Tomahawk, WI, USA) around three main areas on the island of St. Kitts (Figure 1), from March to April 2017. All trapping locations were in urban areas where rats were expected to be passing. Traps were set overnight and checked the following morning. The captured rats were then immediately transported to the RUSVM necropsy facility.

### 2.2. Sample Collection and Rat Identification

Captured rats were euthanized using carbon dioxide gas (CO_2_) and cervical dislocation. This was performed by placing traps containing the rats in a leak-proof plastic bag and filling it with CO_2_. Once the rats were visibly unconscious, they were taken out of the plastic bag and cervical dislocation was performed as secondary euthanasia. Subsequently, blood was collected by open cardiac puncture. We recorded the weight, sex, and body length of each rat. Serum was obtained after centrifugation of whole blood, and stored at −80 °C until used for serological analysis. The rat species was determined by amplification and sequencing of the mitochondrial cytochrome b gene [5]. Sequences were aligned by Molecular Evolutionary Genetic Analysis version 7.0 (MEGA7) [6] and a search of homologous sequences was performed using Basic Local Alignment Search Tool (BLAST) [7].

### 2.3. Serological Analysis

Serum samples were submitted to a commercial laboratory (IDEXX BioAnalytics, Columbia, MO, USA) to detect antibodies against the following agents using a multiplex fluorescent immunoassay 2 (MFI2) panel (Rat Global Serology): cilia-associated respiratory bacillus (CARB), *Clostridium piliforme*, *Mycoplasma pulmonis*, *Encephalitozoon cuniculi*, *Pneumocystis carinii*, Toolan’s H-1 virus (H-1), Hantaan virus (HTNV), infectious diarrhea of infant rats strain of group B rotavirus (IDIR strain of GBR; rat rotavirus), Kilham rat virus (KRV), lymphocytic choriomeningitis virus (LCMV), mouse adenovirus type 1 (MAV1), mouse adenovirus type 2 (MAV2), pneumonia virus of mice (PVM), rat coronavirus/sialodacryoadenitis virus (RCV/SDAV), reovirus type 3 (REO3), rat minute virus (RMV), rat parvovirus (RPV), rat theilovirus (RTV), and Sendai virus (SeV).

## 3. Results

A total of 29 rats were collected from three areas around the island of St. Kitts, of which 22 were tested for antibodies against various rodent pathogens. For the other seven rats, a sufficient amount of serum was not able to be collected for this study. Of those 22 rats for which an adequate amount of serum was available, 12 (54.5%) were males and 10 (45.5%) were females. The rat species were identified as *R. norvegicus* (13/22; 59.1%) and *R. rattus* (9/22; 40.9%).

**Figure 2:**
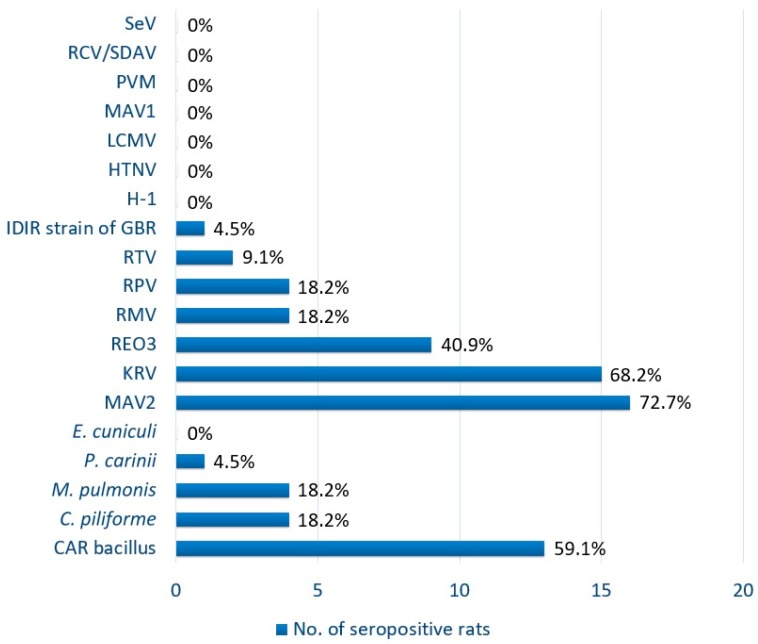
Seroprevalence of selected rodent pathogens in wild rats from St. Kitts, West Indies. CARB, cilia-associated respiratory bacillus; H-1, Toolan’s H-1 virus; HTNV, Hantaan virus; IDIR strain of GBR, infectious diarrhea of infant rats strain of group B rotavirus (rat rotavirus); KRV, Kilham rat virus; LCMV, lymphocytic choriomeningitis virus; MAV1, mouse adenovirus type 1 (strain FL); MAV2, mouse adenovirus type 2 (strain K87); PVM, pneumonia virus of mice; RCV/SDAV, rat coronavirus/sialodacryoadenitis virus; REO3, reovirus type 3; RMV, rat minute virus; RPV, rat parvovirus; RTV, rat theilovirus; SeV, Sendai virus.

Exposure to 11 of 19 (57.9%) pathogens tested in the panel was detected, and 21 of the 22 (95.5%) rats sampled were positive for one or more pathogens tested (Table 1, Figure 2). Of the 22 tested serum samples, 72.7% (16/22; 95% confidence interval (CI): 54.1–91.3) were positive for MAV2, 68.2% (15/22; 95% CI: 48.7–87.7) for KRV, 59.1% (13/22; 95% CI: 38.6–79.6) for CARB, 40.9% (9/22; 95% CI: 20.4–61.4) for REO3, 18.2% (4/22; 95% CI: 2.1–34.3) for *C. piliforme*, *M. pulmonis*, RPV, and RMV, 9.1% (2/22; 95% CI: 0–21.1) for RTV, and 4.5% (1/22; 95% CI: 0–13.2) for *P. carinii* and IDIR strain of GBR (Table 1; Figure 2). No serological evidence of *E. cuniculi*, HTNV, LCMV, THV, MAV1, PVM, RCV/SDAV, and SeV was detected in any serum sample. Antibodies to significant zoonotic pathogens (HTNV and LCMV) were not detected in any of the samples. According to the serological results, one rat was negative for antibodies to all agents tested, and three rats were positive for antibodies to a single pathogen (two MAV2 and one RTV). Four rats were positive for antibodies to two tested pathogens, while 14 rats had antibodies against three or more of the pathogens tested. The highest number of pathogens detected was in a single rat with antibodies to seven pathogens. *R. norvegicus* had significantly higher prevalence of KRV (12/22; *p* ≤ 0.0066) and CARB (11/22; *p* ≤ 0.0073) compared to *R. rattus* (3/22 and 2/22, respectively). Fisher’s exact test was the statistical method used due to the small sample size. There was no association identified between the presence of any pathogen and sex of the rat.

## 4. Discussion

This pilot seroprevalence study provides first evidence of the exposure to rodent pathogens in wild rats in St. Kitts, West Indies. Sampling the wild rodent population in St. Kitts for common pathogens known to affect laboratory rodent colonies would be substantially beneficial in enhancing the current knowledge when implementing routine animal health monitoring protocols with respect to the laboratory rodent colony at RUSVM, in addition to assessing for zoonotic risks to laboratory animal care personnel.

In this study, antibodies against CARB, MAV2, and KRV were detected in over half of the rat serum samples tested. KRV, like H-1, RMV, and RPV, is a parvovirus that is frequently found in laboratory and wild rats that can persist in infected rats and the environment for long periods of time, and rats are a natural host for the virus. Unlike H-1, which has low significance in rats, KRV could tremendously interfere with biomedical research involving several body systems, especially if infection occurred during fetal development [3]. Parvoviruses H-1, RMV, and RPV are asymptomatic in naturally infected rats, compared to KRV, which, although rarely, causes clinical signs such as jaundice, ataxia, and scrotal cyanosis [3,8]. Genetic and/or behavioral differences could be attributed to the increased seroprevalence of KRV in *R. norvegicus*.

The highest seroprevalence was to MAV2 (strain K87), while none of the samples were positive for MAV1 (strain FL). Mouse adenoviruses are rare and asymptomatic in mice and have minimal interference in biomedical research [3,9]. The high seroprevalence observed in this study could be attributed to known seroconversion of a different virus to MAV2 in some rat colonies, and the cross-reactivity of MAV1 antiserum with MAV2 [9].

Seroprevalence of CARB in this study was much higher than that of *M. pulmonis*, which contradicts previous findings of significant correlation and co-infection between the two pathogens [3,10,11]. In addition, we found a positive association between exposure to CARB and the species of rat (*R. norvegicus*), while it was not the case for *M. pulmonis*. Both pathogens are transmitted by aerosol and cause chronic infections in rodents, with dual infection involving both agents causing more severe pulmonary lesions [3]. In a previous study, the authors found that rats in the study had differences in susceptibility to *M. pulmonis* but not to CARB, despite similarities between the two infections [12]. This might be a reason for the large difference in seroprevalences of the two pathogens in our study.

REO3 also had a relatively high seroprevalence, though infected rats are usually asymptomatic and natural infection has not been proven to be linked specifically to interference with biomedical research [13]. Of the three serotypes of reoviruses (1, 2, and 3), type 3 is known to be the most pathogenic to laboratory rodents [3].

Antibodies to HTNV and LCMV were not detected in any of the tested rat serum samples. Both viruses are known to be significant zoonotic pathogens that would be a public health risk [8,14]. Epidemiologically, the majority of HTNV infection is observed in Asia and in its reservoir host, the field mouse (*Apodemus agrarius*). Seoul virus, another hantavirus whose infection occurs worldwide, would be of more concern from a public health perspective, as *R. rattus* and *R. norvegicus* are the main reservoirs [15]. Further evaluation is needed to determine if the antigen used to detect HTNV antibodies in this panel cross-reacts with antibodies to Seoul virus. In addition, it would be essential to screen antibodies against other hantaviruses depending on the local epidemiology.

Natural exposure and infections without any overt disease have been detected in laboratory rodent colonies, and the majority of these infections are caused by opportunists or commensals [3]. Even in the absence of pathogenic effects or clinical disease, colonization with these pathogens may alter biomedical research data [3,8]. Using laboratory animals free from such pathogens contributes to the reliability and reproducibility of results in research studies [8]. In addition to the availability of specific pathogen-free rodents and individually ventilated caging, routine pathogen surveillance and health monitoring protocols in modern research facilities have resulted in lower pathogen prevalence in laboratory rodent colonies [16].

## 5. Conclusions

In this pilot seroprevalence study, we demonstrated evidence of exposure to rodent pathogens in wild rats in St. Kitts. Wild rats harbor a variety of rodent and zoonotic pathogens [1] that could be a source of contamination in laboratory rodent colonies, which may compromise biomedical research data, as well as jeopardize human and animal health [3,8]. Periodic pathogen surveillance in the wild rat population would be beneficial in assessing potential regional zoonotic risks as well as in enhancing the current knowledge when implementing routine animal health monitoring protocols in research and breeding facilities with laboratory rodent colonies.

## Figures and Tables

**Table 1:** Overall summary of rodent pathogens detected in wild rats from St. Kitts, West Indies.

Pathogen	Area A	Area B	Area C	Total (%)
**Bacteria**				
CARB	0/4	2/2	11/16	13/22 (59.1)
*C. piliforme*	1/4	1/2	2/16	4/22 (18.2)
*M. pulmonis*	0/4	0/2	4/16	4/22 (18.2)
**Fungi**				
*E. cuniculi*	0/4	0/2	0/16	0/22 (0)
*P. carinii*	0/4	0/2	1/16	1/22 (4.5)
**Viruses**				
H-1	0/4	0/2	0/16	0/22 (0)
HTNV	0/4	0/2	0/16	0/22 (0)
IDIR strain of GBR	0/4	0/2	1/16	1/22 (4.5)
KRV	0/4	1/2	14/16	15/22 (68.2)
LCMV	0/4	0/2	0/16	0/22 (0)
MAV1	0/4	0/2	0/16	0/22 (0)
MAV2	2/4	1/2	13/16	16/22 (72.7)
PVM	0/4	0/2	0/16	0/22 (0)
RCV/SDAV	0/4	0/2	0/16	0/22 (0)
REO3	0/4	0/2	9/16	9/22 (40.9)
RMV	0/4	1/2	3/16	4/22 (18.2)
RPV	0/4	1/2	3/16	4/22 (18.2)
RTV	1/4	1/2	0/16	2/22 (9.1)
SeV	0/4	0/2	0/16	0/22 (0)

Area A, around the campus of Ross University School of Veterinary Medicine in West Farm, and residential dwellings in Mattingley; Area B, near commercial establishments in downtown Basseterre and Port Zante; Area C, around the campus of the St. Kitts Biomedical Research Foundation (nonhuman primate facility) in Lower Bourryeau Estate. Abbreviations: CARB, cilia-associated respiratory bacillus; H-1, Toolan’s H-1 virus; HTNV, Hantaan virus; IDIR strain of GBR, infectious diarrhea of infant rats strain of group B rotavirus (rat rotavirus); KRV, Kilham rat virus; LCMV, lymphocytic choriomeningitis virus; MAV1, mouse adenovirus type 1 (strain FL); MAV2, mouse adenovirus type 2 (strain K87); PVM, pneumonia virus of mice; RCV/SDAV, rat coronavirus/sialodacryoadenitis virus; REO3, reovirus type 3; RMV, rat minute virus; RPV, rat parvovirus; RTV, rat theilovirus; SeV, Sendai virus.
